# EPIGENETIC PREDICTORS OF LIFESPAN AND HEALTHSPAN

**DOI:** 10.1093/geroni/igz038.130

**Published:** 2019-11-08

**Authors:** Ake T Lu, Steve Horvath

**Affiliations:** 1 Department of Human Genetics, University of California, Los Angeles, Los Angeles, California, United States; 2 Department of Human Genetics, UCLA, LOS ANGELES, California, United States

## Abstract

Capturing aspects of biological age, DNA methylation based biomarkers collectively known as "epigenetic clock" can be used to measure the age of any human tissue, cell type, or fluid that contains DNA. Arguably the strongest predictor of lifespan, DNAmGrimAge, is a composite biomarker comprised of DNAm-based surrogates of plasma proteins and a DNAm-based estimate of smoking pack-years. Large-scale validation studies demonstrate that DNAmGrimAge stands out among existing epigenetic clocks when it comes to predicting time-to-death (P=2.0E-75), time-to-coronary heart disease (P=6.2E-24), and its strong relationship with computed tomography measures of fatty liver, and age-at-menopause (P=1.6E-12). DNAm-based estimates of plasma proteins and telomere length (TL) are attractive as well. For example, a DNAm based estimate of Plasminogen Activator Inhibitor 1 strongly relates to multi-morbidity and a DNAm based estimate of TL outperforms actual TL measurements in predicting lifespan. Overall, these epigenetic biomarkers are expected to find many applications including human anti-aging studies.

